# Understanding the MIND phenotype: macrophage/microglia inflammation in neurocognitive disorders related to human immunodeficiency virus infection

**DOI:** 10.1186/s40169-015-0049-2

**Published:** 2015-02-26

**Authors:** Amanda Brown

**Affiliations:** Johns Hopkins University School of Medicine, 600 North Wolfe Street/Meyer 6-181, Baltimore, MD 21287 USA

**Keywords:** Macrophage polarization, Neuroinflammation, HIV-associated neurocognitive disorder, Microglial activation, Neurodegeneration, Neuronal dysfunction, Osteopontin, Proinflammatory cytokine

## Abstract

Tissue macrophages play important roles in maintaining homeostasis in most organs of the body including the brain where microglia represent the resident phagocytic cells of this compartment. The possibility of one day harnessing macrophage plasticity to treat or ameliorate disorders including obesity, cancer, organ damage, intestinal disorders, neurodegeneration, and cardiovascular disease in which these cells play a role, is a very exciting prospect. Inflammatory signaling is required for regenerative repair, healing, and pathogen clearance functions. However, when the inflammatory response persists in a chronic fashion over an extended period of time, damage to neurons is followed by neuronal injury and dysfunction. Macrophages in the brain are heterogeneous arising from tissues during embryogenesis, and in the adult, from bone marrow derived monocytes that enter through the blood–brain-barrier. While much of our insight regarding macrophage functional subtypes has been garnered through elegant studies in mice, which are amenable to genetic manipulation, far less is known about such cells in human tissues, and particularly in the brain under normal, disease, or injurious conditions. In this regard, non-human primate models for human immunodeficiency virus have been extremely useful for understanding the contribution of bone marrow-derived monocytes in neurological disease and their interaction and impact on the activation state of resident microglia in the brain. This review will focus on what has been learned from the rhesus macaque models about the types of macrophages present in the brains of animals with encephalitis. *In vitro* studies, which have used human blood monocytes differentiated into macrophages to address the question of macrophage subsets in HIV infection will be highlighted. Recent insights on macrophage phenotype and persistent inflammation in the brain in HIV-associated neurocognitive disorder from immunohistochemical studies on human autopsy tissue will be examined.

**Electronic supplementary material:**

The online version of this article (doi:10.1186/s40169-015-0049-2) contains supplementary material, which is available to authorized users.

## Introduction

The possibility of one day harnessing macrophage plasticity to treat or ameliorate disorders including obesity, cancer, organ damage, intestinal disorders, neurodegeneration and cardiovascular disease in which these cells play a role, is a very exciting prospect. With the elegant studies done in mice, much has been learned about the ontogeny of tissue macrophages, their ability for local proliferation and the recruitment of monocytes from the adult bone marrow under normal homeostatic conditions versus in disease states or in the case of injury [1-3]. In this review, some of the seminal findings from mice on monocyte-macrophage development will be highlighted and then the focus turned on the nervous system with an emphasis on recent findings on macrophage phenotype in non-human primates and humans as it relates to human immunodeficiency type 1 (HIV)-mediated neuropathogenesis.

## Review

Macrophages were first identified in 1841 as inflammatory corpuscles in damaged brain, as foam cells by Virchow in 1846, stained by Weigert and Golgi in 1873, and its key function as a mediator of phagocytosis was reported by Metchnikoff in 1882 [4]. The central nervous system, and particularly the brain, represents the farthest reaches of the frontier, as any future translational therapeutic approaches must, based on our current understanding, surmount the blood–brain-barrier, penetrate into the brain parenchyma, and localize to the affected site in order to have a beneficial effect.

It is now well established, based on a series of recent elegant studies using mouse genetics, lineage fate mapping techniques, and transcription profiling, that embryonic monocytes give rise to the majority of tissue macrophages in an adult animal except for in the intestine, heart, and skin [1-3]. Moreover, these tissue macrophages are capable of self-renewal. However, in the context of neuronal injury and infection, patrolling monocytes/macrophages from the bone marrow will invade the brain. In this regard, trafficking of CCR2 expressing monocytes to the brain in a model of Alzheimer’s disease, plays a key role in ameliorating the toxic effect of amyloid accumulation [5]. In the adult animal, myeloid cells develop in the bone marrow from a CD34+ hematopoietic stem cell that gives rise to a common myeloid progenitor that can migrate into tissues [6,7]. Turnover studies in mice suggest that monocytes circulate in the blood for 1–3 days before entering tissues where, depending on signals received from local resident macrophages [1], they differentiate into mature cells with distinct morphology and function. Whether different subpopulations of monocytes give rise to specific types of tissue macrophages is not currently fully understood, but there is agreement that the presence of the fractalkine receptor CX_3_CR1 marks inflammatory monocytes that migrate into tissues in response to its ligand [8,9].

At least three distinct populations of monocytes have been identified in human blood based on the expression of the cell surface receptors: 1) CD14^hi^CD16^−^ (classical) which also express CCR2^hi^, CX_3_CR1^lo^ and represent 80-90% of total monocytes, 2) the CD14^+^CD16^hi^ (non-classical), and 3) the CD14^hi^CD16^lo^ (intermediate) proinflammatory monocytes that also express CX_3_CR1, and low levels of CCR2, and are found in the blood at 5-10% [10]. This latter population is preferentially infected by in HIV [11] and infiltrates the brain during infection [12,13]. Moreover, transcriptional profiling suggests that distinct genetic programs distinguish the three subsets [14,15]. What we do not yet know is whether these subpopulations can be reprogrammed into different subtypes. *In vitro,* and likely in microenvironments within tissue compartments, specific cytokines can polarize monocytes to develop along different effector pathways that have been called M1 or M2, analogous to the nomenclature used for T-cell subsets [16-18]. Several excellent reviews discuss the discovery and spectrum of phenotypes and functional characteristics of these subtypes of macrophages [18,19].

In addition to inflammatory-directed polarization, the determinants of macrophage morphology and function may in part, be governed by the cells in the microenvironment with which macrophages interact. For example, a subtype of macrophage found in the intestine, the muscularis macrophages, associate very tightly with enteric neurons to help regulate intestinal peristalsis [20]. Analogous to the microglia-neuron regulatory signaling mechanism using fractalkine ligand on neurons and fractalkine receptor on microglia, muscularis macrophages secrete bone morphogenetic protein 2 (BMP2), which activates the BMP receptor on enteric neurons [20]. Microglia, the resident macrophages in the brain, assume an ameboid shape when engaged in phagocytic functions. Microglia display an extensive ramified morphology under normal homeostatic conditions during which they continually make contact through extended finger-like projections to neurons in their vicinity [21,22]. Microglia also play critical roles in shaping neuronal networks during development, and in the adult animal by modulating synaptic transmission [21,23].

Within the brain, macrophage phenotype varies with their location in this tissue. Perivascular macrophages, as the name implies, are located intimately with vessels and enter from the blood circulation into the brain at a low level during normal conditions, and at higher frequency in the context of damage or invasion of the brain by a pathogen. Choroid plexus macrophages and meningeal macrophages, which are closely associated with the meninges, the thin blood vessels that line the brain, express MHC class II and costimulatory molecules. Parenchymal macrophages include the microglia population and cell surface markers such as CD68, Iba1, and CD163 stain both cell types [24]. Moreover, it is possible that infiltrating macrophages that move deeper into the parenchyma are able to do so because the appropriate transcriptional program has been initiated and recognized within the local microenvironment. Indeed, blood monocytes can home to the brain when microglia are experimentally depleted in mice [25]. However, the bone marrow-derived microglia are not able to penetrate deeply into the brain parenchyma, which suggests the possibility that they lack the genetic instructions to do so and/or that they do not receive the proper secondary signals perhaps because they are not in the correct location [25].

Traffic across the blood–brain-barrier is strictly regulated, but many pathogens are able to gain entry through monocytes, which are able to extravasate across. HIV enters the body through mucosal sites where it infects resident macrophages, dendritic cells, and CD4+ T-cells at these sites leading to dissemination throughout the lymphatic system. HIV enters the brain rapidly after infection. Entry into the brain was shown in a case of human iatrogenic transmission, and to occur within three to fifteen days depending on the experimental monkey model examined [26-29]. In the absence of treatment, encephalitis occurs in ~20-30% of infected individuals, which is characterized pathologically by the presence of multinucleated giant cells, microglia nodules, microgliosis, astrocytosis, and abundant CD68+ macrophages staining for HIV antigens [30]. As not all HIV-infected individuals develop CNS dysfunction, there is a role for host genetics in conferring susceptibility or resistance to the development of cognitive impairment [31].

The simian immunodeficiency virus (SIV) non-human primate models of HIV infection have been invaluable in adding to our understanding of the central role of macrophages as initiators of an inflammatory cascade, which ultimately results in neuronal damage and dysfunction [32,33]. Analyses of blood monocytes from infected individuals with HIV-associated dementia detected an expansion of the CD14 + CD16+ subset [12], which was later confirmed in SIV-infected monkeys. Indeed, monocyte turnover in the bone marrow is a better predictor of progression to AIDS than CD4+ T-cell count and plasma viral load [34,35]. A very recent set of studies suggests a mechanism for CCR2/CCL2 signaling in the recruitment and trafficking of CD14 + CD16+ monocytes into the brain in HIV-associated cognitive impairment [36,37]. The CD14 + CD16+ monocytes that crossed an artificial blood–brain-barrier model as well as those found in the CSF of individuals with HIV-associated cognitive impairment, were found to preferentially express CCR2 [37]. Moreover, antibody against the tight junction protein JAM-A and adhesion molecule ALCAM was able to block the accumulation of CD14 + CD16+ monocytes [38]. The macrophage marker CD163 is present on CD14 + CD16+ blood monocytes expanded in SIV encephalitis and these cells are believed to be the bone marrow derived CD34+ precursors of the CD163+ perivascular macrophages seen in the brain [39-41]. Moreover, CD68 + CD163+ macrophages accumulate in the CNS in SIV-infected monkeys and in human brain tissue from HIV-infected individuals [34,39-42]. In SIV encephalitis, productively infected CD14 + CD16 + CD163 + CD45hi perivascular macrophages are abundant. The myeloid marker MAC387 is found on BrdU+ monocytes/macrophages in SIV-infected animals in the early stages of CNS invasion only when inflammation is abundant [43]. In contrast, in chronic lesions CD68 + CD163+ macs are most highly represented in SIV-infected animals and in HIV-infected human with encephalitis [34,39]. Moreover, MAC387 + CD163-CD68-CCR2- macrophages do not appear to be productively infected with HIV. Multinucleated giant cells, which are the hallmark of fulminant HIV replication in macrophages, expressed CCR2 and CD68 [43]. Collectively, these data suggest the possibility that HIV-infected and uninfected M1-type macrophages may be present early, and as the host attempts to downregulate the immune response, M2-type macrophages become more abundant [44]. In *in vitro* monocyte-derived macrophages (MDM), HIV replication in M1 and M2 macrophages is reduced, however the extent of the inhibition varies with the stimuli used [45,46]. Microarray analyses on HIV-infected MDM have shown that the production of proinflammatory cytokines is increased through a TLR-independent pathway suggesting that HIV infection induces an M1-type milieu [47]. A proteomic study suggested that M1-HIV infected macrophages cocultured with T-regulatory cells can shift to an M2 phenotype, which was associated with neuroprotection [48]. Further study of the potential role played by M1 and M2 type macrophages in HIV CNS infection is required.

There are currently two approaches for investigating the contribution of human macrophage phenotype as it relates to HIV infection: 1) with *in vitro* culture models based on the isolation and differentiation of monocytes isolated from the blood (monocyte-derived macrophages or MDM) and 2) immunophenotyping using brain tissue obtained at autopsy. Monocyte isolation methods vary from low purity using adherence to plastic, to density gradient centrifugation, elutriation, or the use of positive or negative immunomagnetic bead selection for example for CD14+ cells. Each of these methods has their advantages and drawbacks, which relate to the level of purity, yield, and the inadvertent induction of cellular activation. Culture conditions can also vary widely and may make comparisons between laboratories difficult. This topic has been recently addressed and recommendations made [19].

We have used a standardized culture model for several years in conjunction with a recombinant HIV that encodes the enhanced green fluorescent (GFP) gene in a portion of the viral genome that is expressed early during infection [49-51]. Using flow cytometric analyses, we identified a consensus surface activation marker signature (SAMS), CD14^+^CD69^+^CD86^+^CD68^lo^ on a subpopulation of MDM in which HIV replication was active [52]. Interestingly, the presence of CD69, but not CD14 or CD86 on the cell surface was dependent on the expression of the viral protein Nef in the infected macrophage. Nef is essential for disease pathogenesis *in vivo*, for robust replication in primary T-cells and macrophages *in vitro,* and helps HIV to evade the immune response through multiple mechanisms [53-57]. Induction of CD69 expression on murine macrophages treated with LPS, TNF-α or IFN-γ and LPS has been reported, however relatively little is currently known regarding the function of CD69 on this cell type [58]. One study suggests that CD69 may have a role in downregulating immune responses through TGF-γ [59]. CD86 is a marker for M1-type macrophages and its presence together with CD69 suggests that these cells are highly activated. We understand what may at first seem paradoxical, that HIV would prefer to replicate in an activated cell, is that the virus has evolved to use host proteins as cofactors in the essential steps of reverse transcription, and transcription of the integrated proviral DNA. These cofactors are present at sufficient concentrations only in stimulated cells. At least in this *in vitro* model, both HIV-dependent and –independent mechanisms are involved in the regulation of expression of these innate inflammatory surface proteins. It remains to be determined whether the phenotype identified will be recapitulated in human tissues *in vivo*, however CD14 + CD16 + CD69+ monocyte/macrophages were previously reported to be expanded in the brain during HIV infection [12].

In regions of the world where therapy is widely available, the incidence of HIV encephalitis and full-blown dementia has greatly diminished [60,61]. However, the prevalence of milder forms of HIV-associated cognitive impairment has increased as people live longer with the infection. Importantly, comorbidities including aging, illicit drug use, exposure to antiretrovirals, cardiovascular disease, and insulin resistance could potentially contribute to cognitive impairment [62,63]. A major goal of the field is to identify the neuropathogenic mechanisms leading to the persistence of neuronal injury and dysfunction in HIV infection despite complete suppression of viral replication in the periphery. Much of the macrophage phenotype data that currently exists utilized autopsy tissue that predated the widespread use of antiretroviral inhibitors in the developed world.

Hence, there is a need to identify biological markers that could help predict risk for the development of cognitive impairment, and would serve a predictive function in assessing the efficacy of current treatments and for novel therapeutics in development. In this regard, a recent study suggests that individuals with asymptomatic neurocognitive impairment (ANI) have a 2-6-fold increased risk of progressing to further cognitive decline [64]. One recurring and prominent feature is the persistence of immune activation. Proinflammatory markers including IL-6, sCD14, sCD163 remain elevated in those on successful anti-viral therapy [63,65]. In part, the persistence of these factors is due to early HIV-mediated damage to the gastrointestinal tract in which microbial cell wall components are released into the circulation [66]. Moreover, the presence of these latter inflammatory mediators is associated with cognitive impairment in HIV [67].

Osteopontin (OPN) is a proinflammatory cytokine first described as early T-cell activation marker 1 that is expressed in several cell types including T-cells and tissue macrophages [68]. OPN was detected as a cytokine that was highly upregulated in the brains of monkeys with SIV encephalitis and later shown to also be elevated in the CSF and brain of individuals with HIV-associated neurocognitive disorder [69,70]. Interestingly, OPN enhances HIV replication in macrophages by 50% through mechanisms that involve activation of the NF-κB responsive viral promoter, perhaps through integrin receptors and also by promoting cell-to-cell adhesion, which facilitates viral spread [69,71]. Interestingly, OPN levels in the plasma remain high despite undetectable HIV viral load, supporting other observations that inflammatory processes remain active [72]. Both HIV-infected and uninfected inflammatory monocytes have been shown to be key contributors to the inflammation seen in the brain [73]. We hypothesized that these cells were the major source for OPN in the brain of HIV-infected individuals. However, a recent immunohistochemical study on autopsy brain tissue suggests that astrocytes are also a source of OPN, although no significant differences in expression were seen between the HIV-infected and HIV-infected with cognitive impairment groups [74]. Unexpectedly, however, significant expression of OPN was detected in cortical neurons of brain tissue from those with HIV-associated cognitive impairment [74].

**Figure 1:**
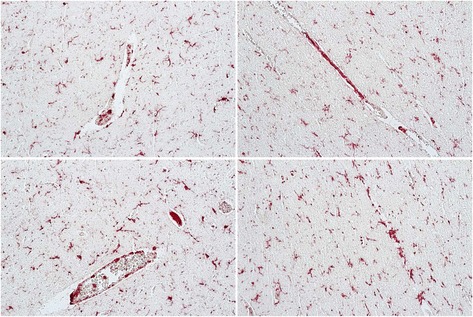
**Abundant Iba1/AIF-1 positive parenchymal and perivascular macrophage/microglia (red color) costain for osteopontin (OPN) (brown color) expression in tissue from the occipital lobe of an HIV-infected individual with asymptomatic neurocognitive impairment (ANI).** Paraffinembedded human autopsy tissue from the occipital lobe (National NeuroAIDS Tissue Consortium). Antigen retrieval was performed in citric acid buffer pH 6.0 and slides were stained sequentially with rabbit polyclonal antisera against Iba1/AIF-1 (SIGMA) overnight at 4°C followed by incubation with goat-anti-rabbitalkaline phosphatase (AP) secondary for 1 hr at room temperature and developed with permanent FastRed Quanto (ThermoFisher) (red color). Slides were then incubated with mouse monoclonal antibody to OPN (MAB194, Maine Biotechnology) at room temperature for 2 hrs followed by goat anti-mouse-horse radish peroxidase for 1 hr and developed with 3,3’-diaminobenzidine (brown color). Images were taken on an Axio Observer A1 inverted microscope (Zeiss) at 20x magnification. Adjustment of the image brightness, contrast and sharpness was performed with Adobe Photoshop 5.5 using the same settings for each image.

**Figure 2:**
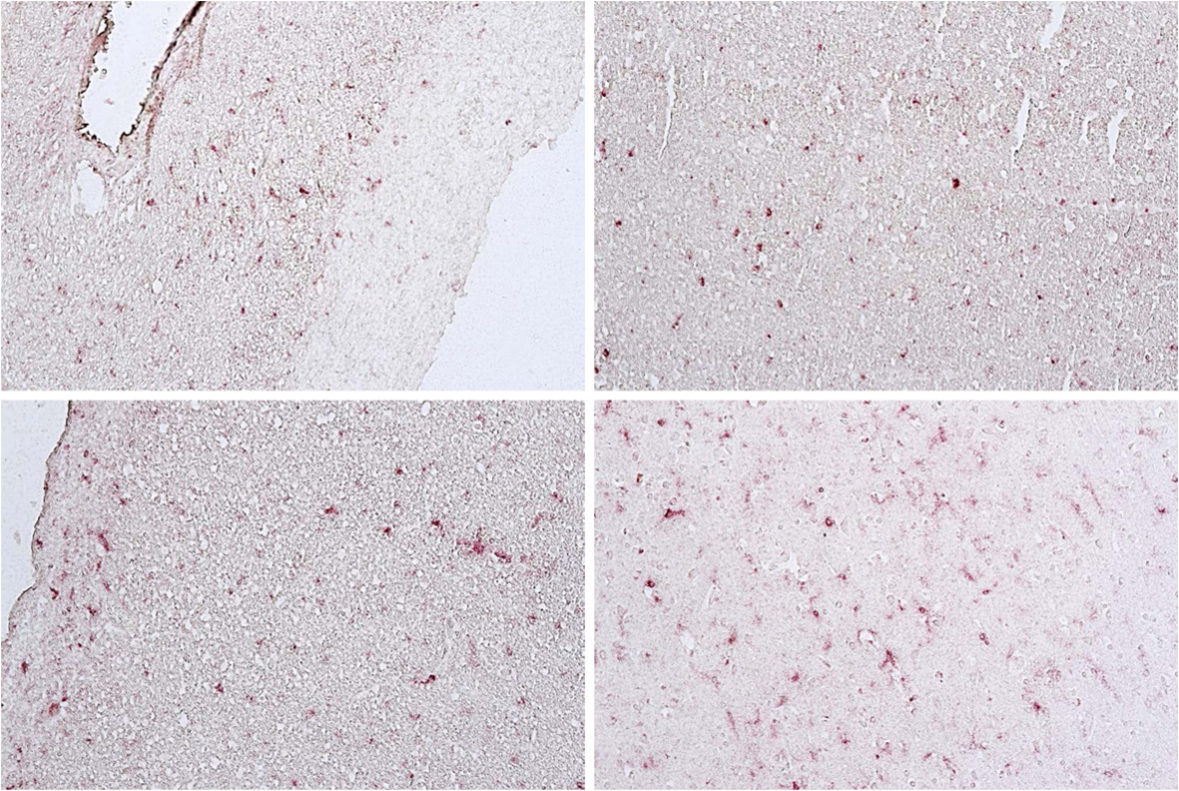
**Iba1/AIF-1 positive parenchymal macrophage/microglia (red color) costain for osteopontin (OPN) (brown color) expression in tissue from the occipital lobe of an HIV-infected individual with minor neurocognitive disorder (MND). **Microglia with ameboid morphology are more abundant than cells with a ramified phenotype.

**Figure 3:**
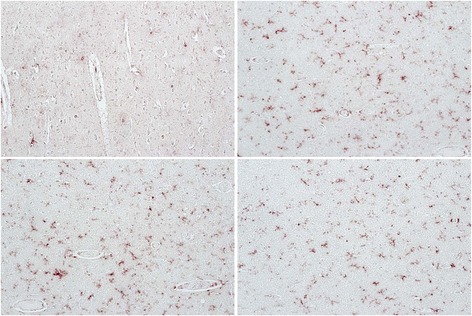
**Abundant expression of osteopontin (brown color) in Iba1/AIF-1 positive parenchymal and perivascular macrophage/microglia (red color) in tissue from the occipital lobe of an HIV-infected individual with HIV-associated dementia (HAD).** Osteopontin in HIV-infected HAD cases were significantly elevated compared to normal controls [74].

HIV-associated neurocognitive disorders (HAND) is an umbrella term to describe three levels of neurocognitive dysfunction: asymptomatic neurocognitive impairment (ANI) in which individuals show deficits (greater than one standard deviation) in two or more cognitive domains, but no impairment of activities of daily living (ADL); minor neurocognitive disorder has the same level of impairment as ANI, but ADLs are mildly affected and HIV-associated dementia (HAD) represents the most severe form of impairment in which deficits in two or more cognitive domains is greater than two standard deviations and there are a severe impact on ADLs [75,76]. In tissue from an HIV-infected individual with ANI, with very low viral load in the CSF (19 RNA copies/ml) or plasma (249 RNA copies/ml) who had been on antiretrovirals, an abundance of double-stained Iba-1/OPN perivascular macrophages and microglia and parenchymal microglia were detected (Figure 1). In a case with MND, the microglia appear to be predominantly of the ameboid type with few ramified processes (Figure 2). This patient at death despite being on therapy had a plasma viral load of >48,000 copies/ml, a CSF load of 148 copies/ml and was severely immunosuppressed with a CD4 T-cell count of 10. A case with HAD displayed microgliosis with abundant ameboid and ramified microglia in the parenchyma, and a high level of OPN expression (Figure 3). This individual at death had a high plasma viral load of ~40,000 copies/ml, a CSF load of 2747 copies/ml but a CD4 T-cell level of 299, not indicative of immunosuppression. Another study also found, as detected by the markers CD16, CD163, HLA-DR, and GFAP, that for macrophages/microglia and astrocytes, elevated levels of inflammation in the brain remained a common feature in HIV-infected individuals without evidence of encephalitis or productive viral replication in the brain [77]. Together these results highlight the variation in microglia activation that persists in the brain at the individual level and the need for reliable plasma and/or CSF markers that would allow clinicians to track smoldering CNS inflammation.

## Conclusion

Much research remains to understand the communication between macrophages and other cells in the CNS. We need to identify the key inflammatory signals that are transmitted from the periphery to the brain and vice versa, which regulate homeostasis [21,78]. Comorbidities and host genetics are also important contributors that must be teased out and animal models will continue to play an essential role in these investigations. At the same time, human plasma and CSF have shown promise in uncovering the identity of potential biomarkers [79-85]. In the future, with regards to modulating macrophage phenotype in the CNS to counter neurotoxicity and promote regeneration [86], perhaps targeted delivery to the nasal compartment may provide an alternative portal to the brain [87]. We should all get excited about the small, yet significant steps that advance our understanding of macrophage phenotype, such that one day, we can make the giant leap to tackle disease and dysfunction in the MIND.

## Abbreviations

GFP: Green fluorescent protein
HIV: Human immunodeficiency virus type 1
CSF: Cerebrospinal fluid
CNS: Central nervous system
Iba 1: Ionized calcium binding adaptor protein 1
OPN: Osteopontin
TLR: Toll-like receptor
MDM: Monocyte-derived macrophage
ANI: Asymptomatic neurocognitive impairment
MND: Minor neurocognitive disorder
HAD: HIV-associated dementia
HAND: HIV-associated neurocognitive disorders
SIV: simian immunodeficiency virus
ADLs: Activities of daily living
